# Psychotropic Polypharmacy in the US Pediatric Population: A Methodologic Critique and Commentary

**DOI:** 10.3389/fpsyt.2021.644741

**Published:** 2021-06-14

**Authors:** Julie M. Zito, Yue Zhu, Daniel J. Safer

**Affiliations:** ^1^Department of Pharmaceutical Health Services Research, School of Pharmacy, Baltimore, MD, United States; ^2^Department of Psychiatry, School of Medicine, University of Maryland, Baltimore, MD, United States; ^3^Department of Epidemiology, School of Public Health, George Washington University, Washington, DC, United States; ^4^Department of Psychiatry, The Johns Hopkins Hospital, Johns Hopkins Medicine, Baltimore, MD, United States

**Keywords:** polypharmacy, pediatric, concomitant psychotropic, children, adolescents, multiple medications or concurrent psychotropics

## Abstract

**Background:** Psychotropic concomitant medication use for the treatment of youth with emotional and behavioral disorders has grown significantly in the U.S. over the past 25 years. The use of pharmacy claims to analyze these trends requires the following: age of the selected population, overlapping days of use, and precision of the outcome itself. This review will also address the gaps in reporting of pediatric psychotropic polypharmacy.

**Methods:** An electronic literature search was undertaken for the period 2000 through 2020 using keywords such as “pediatric,” “concomitant,” “polypharmacy,” “multiple medications,” and “concurrent psychotropic”; Relevant references in textbooks were also used. Only English language and U.S. studies were included, resulting in 35 inter-class studies.

**Results:** Studies were organized into seven groups according to data sources and clinical topics: (1) population surveys; (2a) multi-state publicly insured populations; (2b) single/two state studies; (3) privately insured populations; (4) diagnosed populations; (5) foster care populations; (6) special settings. Across 20 years it is apparent that pediatric psychotropic polypharmacy affects substantially more children and adolescents today than had been the case. As many as 300,000 youth now receive 3 or more classes concomitantly. The duration of concomitant use is relatively long, e.g., 69–89% of annual medicated days. Finally, more adverse event reports were associated with 3-class compared with 2-class drug regimens.

**Discussion:** Factors that contribute to the growth of pediatric psychotropic polypharmacy include: (1) predominance of the biological model in psychiatric practice; (2) invalid assumptions on efficacy of combinations, (3) limited professional awareness of metabolic and neurological adverse drug events, and (4) infrequent use of appropriate deprescribing.

**Conclusion:** A review of publications documenting U.S. pediatric psychotropic polypharmacy written over the last 20 years supports the need to standardize the methodologies used. The design of population-based studies should maximize information on the number of youth receiving regimens of 3-, 4-, and 5 or more concomitant classes and the duration of such use. Next, far more post-marketing research is needed to address the effectiveness, safety and tolerability of complex drug regimens prescribed for youngsters.

## Introduction

Of U.S. youth less than age 20 years, 21.9% used a prescription drug in the past month according to a recent federal population survey by Hales et al. (1). Furthermore, 39% of these youth used 2 or more prescription drugs of *any* therapeutic class in the previous month. While the prevalence of many therapeutic classes of drugs was stable across the 15 years surveyed, there were prominent increases for several classes. Particularly more widely prescribed were psychotropics used to treat the emotional and behavioral disorders of youth. These included ADHD medications, particularly amphetamine type stimulants, as well as antipsychotics and alpha-adrenergic agents. Unfortunately, the survey authors (1) did not address concomitant use of 2 or more psychotropics, i.e., polypharmacy.

Compared to youth, research on adult polypharmacy in psychiatry has received prominent attention for many years, particularly for adults with serious chronic conditions such as schizophrenia and bipolar disorder (2, 3). The prevalence of 2 or more concomitant classes involved as many as 60% of adult outpatient visits to psychiatrists in 2006 (4).

The definition of polypharmacy varies depending on the parameters measured: the length of overlapping days of exposure and the width of the period assessed (5). Data sources for polypharmacy include population surveys as well as claims-based analyses. Population-based surveys typically measure health care services per 100 eligible persons, often derived from physician office visits. Survey methods typically measure concomitant use as a point prevalence at a single point per year in a population-based model (4). By contrast, period polypharmacy prevalence is more common in administrative claims studies where annual datasets are available to provide a wider window for measurement.

Outcome measures include two types of polypharmacy: within class, e.g., 2 concomitant antipsychotics, and inter-class (multi-class), e.g., concomitant antipsychotic and antidepressant. Within class antipsychotic polypharmacy has been featured in many pediatric studies (6, 7) presumably because it raises concerns with respect to treatment emergent risk, especially for metabolic adverse effects (8, 9). For simplicity of presentation, the most commonly used definition of psychotropic polypharmacy is the use of 2 or more psychiatric medications in the same patient (10).

Medicaid administration programs have sought to reduce the overprescribing of antipsychotics and other psychotropics in children and adolescents, especially foster care youth in response to government reports on overuse (11, 12). As a consequence, state Medicaid oversight programs have produced research showing reduced antipsychotic usage in children (13, 14). The administrative claims data of large populations covered by health insurance have been frequently used to assess inter-class polypharmacy and such studies may feature a single year or multi-year trend analysis. Similarly, all enrolled youth may be represented or youth in a particular subgroup, e.g., foster care youth (15).

This review features inter-class psychotropic polypharmacy for the treatment of youth (16–18). More specifically, the review aims to support administrative claims study methods to:

1. Increase precision in the outcome of polypharmacy beyond “2 or more concomitant drugs” so that 3, 4, and 5 or more class (drug) regimens are reported in terms of the *number and percent of youth as a proportion of psychotropic medicated youth* in a year (19).
2. Standardize methods to:
  - Measure overlapping medication days for 60 or 90 or more days to avoid counting unintentional polypharmacy caused by switching from one drug to another (18, 20).
  - Restrict the denominator of the outcome to all psychotropic medicated youth so as to avoid readers' potential to dismiss low risks, e.g., 20/100,000 (0.02%) enrollees vs. 20/100 (20%) medicated youth.
  - Target meaningful subgroups, e.g., selecting children with autism spectrum disorder (21, 22) or focusing on foster care youth, a high-risk vulnerable population (23, 24).

## Methods

**Figure 1:**
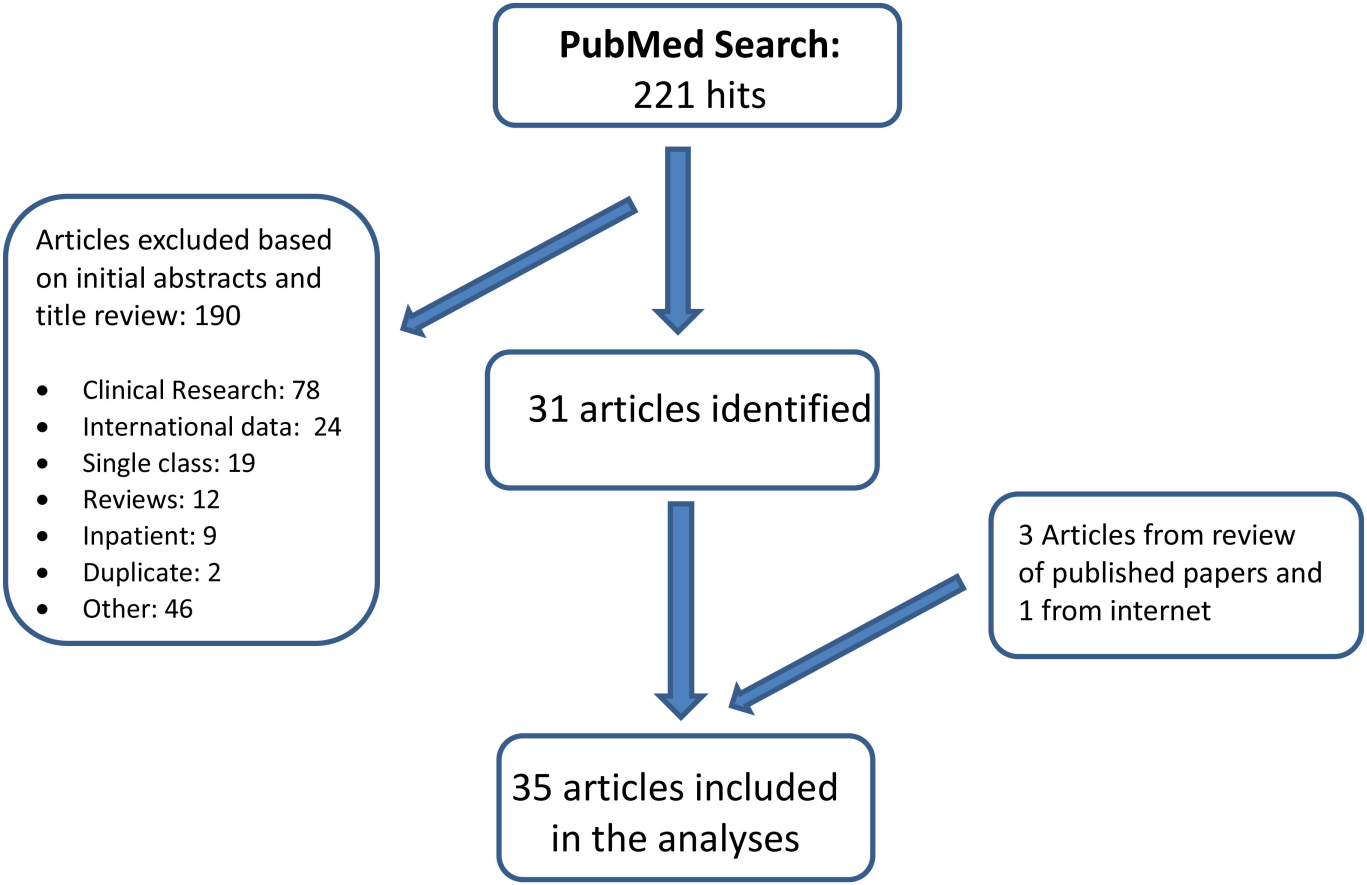
Flow chart of the review process.

A PubMed literature review for the period January 1, 2000-December 31, 2020 was undertaken. Keywords included: Psychotropic OR Psychotropic polypharmacy OR Psychiatric polypharmacy OR Antipsychotics OR Stimulants OR Pharmacotherapy OR Psychotropic medication OR Psychopharmacology; Concomitant OR Concurrent OR Multiple OR Polypharmacy OR Multiclass; Child OR Adolescent OR Youth OR Pediatric; papers were restricted to the English language and U.S. population. In addition, many review papers were scanned for references on quantitative analyses of polypharmacy that may not have been identified in our computerized search. The search results were validated using Embase search. Figure 1 illustrates the search process. We selected 35 papers with quantitative analysis on pediatric psychotropic inter-class polypharmacy for this review. These studies are population-based, mainly relying on either federal physician office visit surveys, parent surveys or administrative drug payment claims.

## Results

Summaries of pediatric psychotropic polypharmacy studies were organized by data source into tables for 7 groups from the latest to the earliest across 20+ years from: (1) Federal and other health care treatment surveys; (2a) MedicaidAnalytic eXtracts (MAX) data for national or multistate analyses; (2b) Single or two state comparisons of publicly funded programs; (3) Privately insured populations; (4) Studies featuring a specific clinician-diagnosed subgroup; (5) The foster care population; and (6) Special treatment settings. Tables 1–6 briefly capture data sources, design, selected populations, critical measurements, and polypharmacy outcome. Many studies fit more than one category but appear only on the most appropriate table.

**Table 1:** Federal and other population-based surveys on pediatric psychotropic polypharmacy.

**Data source, Study period, References**	**Age, years**	**Other**	**No. psychotropic concomitants**	**Point prevalence, denominator**	**Outcome**
MEPS, 1999–2015 Zhang et al. (19)	0–17	3 periods, 1999–2015, parent reported, trends	≥3 classes	0–17 y/o with any psychotropic dispensing	In 2015, nearly 300,000 youth received ≥3 classes concomitantly, a doubling in 12 years
NAMCS/NAHMCS, 2003–2010 Burcu et al. (25)	6–19	Any behavioral diagnostic code (312–314) excluding serious conditions approved for antipsychotic use	Antipsychotic + 1 or ≥2 concomitant classes	6–19 y/o with any prescribed antipsychotic	85% with ADHD diagnosis; 1 concomitant + ATP = 50.7%; 2 concomitants + ATP = 39.1%
NAMCS, 2006–2015; NHAMCS, 2006–2011 Girand et al. (26)	2–24	ADHD diagnosed	≥2 ADHD medications alone; ≥2 ADHD medication + other psychotropics	2–24 y/o with any prescribed ADHD medication	≥2 ADHD meds: 16.8–20.5% ≥2 ADHD + other psychotropic classes: 26.0–40.7%
Community pharmacy-based parent survey Hilt et al. (27)	3–17	Is polypharmacy associated with more adverse drug events? *N* = 1,347 Parent reports of any psychotropic dispensing.	2 classes; ≥3 classes concomitantly	*N* = 1,348 youth w/ any psychotropic dispensing	Compared with montherapy: 2 classes had 17% increase in likelihood of ^*^ADEs;≥3 classes had 38% increase in ^*^ADEs
NAMCS, 1996–2007 Comer et al. (16)	6–17	Any prescribed psychotropics, trends	≥2 classes	6–17 y/o with any prescribed psychotropic	From 14.3 to 20.2% across 11 years
NAMCS, 1993–1998 Bhatari et al. (28)	0–17	Stimulant users, trends	Stimulant + ≥1 psychotropics	0–17 y/o with any prescribed psychotropic	2.9 to 6.9 to 14.7% of stimulant users had ≥1 other psychotropics

* *ADEs, Adverse Drug Events*.

### Federal and Other Population-Based Surveys on Pediatric Psychotropic Polypharmacy

Table 1 identifies key characteristics for comparison of polypharmacy outcomes in 6 studies with increased growth starting in the early ‘90s (28). Major conclusions include: First, Zhang, dosReis et al. (19) showed that across 22 years, the continued growth of regimens of 3 or more concomitant psychotropic classes through 2015 was unmistakable, affecting nearly 300,000 youth treated with complex psychotropic medication regimens (19). Treatment for ADHD, even without comorbidities, is common among complex regimens of U.S. youth (25), often with an antipsychotic and stimulant, a combination with questionable pharmacologic rationale (51). Second, in a 2–24-year-old population of ADHD medication users, recent data showed use of ≥2 ADHD medications (stimulant, atomoxetine, or alpha-agonist) grew from 16.8 to 20.5%, while the much larger pool of ADHD medicated youth received prescriptions for ≥2 other psychotropic classes concomitantly [e.g., antipsychotics and selective serotonin reuptake inhibitors (SSRIs)] and grew during that period from 26.0 to 40.7%. Moreover, the majority of youth in that study were 6–18 years old and psychotropic polypharmacy comprised 73.1% compared with 26.9% for other age groups [2–5 and 19–24-year olds together (26)]. Third, in Hilt et al. (27), parent reports revealed a significantly greater association of adverse drug event reports with 3-drug regimens compared with 2-drug regimens (27). This survey reconfirms the relationship between complex regimens and increased risk of adverse drug events (52). Taken together, all six studies support the need for robust evidence to show the benefit/risk balance in large study cohorts with rigorous methods to assess diagnosis by research standards, monitor drug consumption and measure functional outcomes. Examples include large simple (pragmatic) trials in community treated youth populations to reduce unnecessary treatment and the adverse drug events accompanying that use (53).

### Polypharmacy Studies of Publicly Funded Programs

#### Pediatric Psychotropic Polypharmacy Studies of Publicly Funded Programs Using Medicaid Analytic eXtract (MAX) Data

Table 2a lists 3 studies that analyzed multistate data to provide generalizable Medicaid findings across broad regions of the country. Major assessments from these studies involve the number of classes for outcome and the length of overlap to define polypharmacy. First, the most recent MAX study by Saucedo et al. (29) has outcomes measured in a convenient metric: those with any polypharmacy, whether within or inter-class and those with inter-class only. The outcome showed any 2 or more concomitants (within or inter-class) grew from 21.2% (1999) to 27.3% (2010) across 12 years, a growth of 146,807–189,048 youth among those <18 years old who had any psychotropic dispensing. The vast majority (89.4%) of concomitant use was inter-class rather than within class. Had the data included precise information on 3-class and 4-class concomitant growth, perhaps a stronger case could be made to bring new research on the effectiveness and safety of these common, largely off-label regimens. Second, Chen et al. (30) illustrated the impact of varying the length of overlapping days on 2 or more concomitant classes: longer overlaps decreased the pool identified as having polypharmacy regimens. Widening the prescription overlap from 14 to 30 to 60 or more days reduced polypharmacy from 28.8 to 27.2 to 20.9%. For 60-day overlaps, the overall result is that more than 25% fewer youths are identified, and the captured population is unlikely to include unintentional polypharmacy, i.e., switching drugs. Third, Kreider et al. (31) assessed 6–18-year olds who had continuous annual enrollment and 14 or more overlapping days, but the outcome was limited to pairs of concomitants which does not provide a clear profile of the percentages of youth with 3-, 4-, or 5 or more concomitant classes.

**TABLE 2a:** Pediatric psychotropic polypharmacy studies of publicly funded programs using medicaid analytic eXtract (MAX) data.

**Data source, Study period, References**	**Age, years**	**Other**	**Psychotropic concomitants**	**Overlappingdays**	**Outcome**
1999–2010, 29 states (MAX), Soria Saucedo et al. (29)	0–17	*N* = 692,485 with a psychotropic dispensing, 12 year trend,	≥2 within or Interclass	≥45	21.2% (1999) to 27.3% (2010) for any concomitants, within or interclass. 89% of concomitant use is interclass. ~200,000 youth with ≥2 concomitants in 2010.
2005, 4 large states (MAX), assesses impact of length of overlap re number and % of medicated youth Chen et al. (30)	6–18	*N* = 282,910 with a psychotropic dispensing	≥2 interclass	≥14 ≥30 ≥60	≥14 = 28.8% (81,478) ≥30 = 27.2% (76,951) ≥60 = 20.9% (59,128) Illustrates the impact of avoiding unintentional polypharmacy, i.e., switching.
2004–2008, 42 states (MAX), Kreider et al. (31)	6–18	*N* = 490,000 children; *N* = 540,000 adolescentscontinuous annual enrollees, with a psychotropic class & atypical antipsychotic, 5 year trend,	Inter-class Pairs w/antipsychotic	≥14	Pairs of concomitants: stimulant + ATP = 22.4%;ATD + ATP = 31.7%; mood stabilizer + ATP = 52.1%. Duration of concomitant pairs affected 69–89% of annual medicated days.

#### Single/Two State Medicaid Pediatric Psychotropic Polypharmacy Studies Using State-Based Data

Table 2b lists 7 studies derived from state-specific datasets which are often less costly to acquire and offer potential advantages in terms of providing information to local quality assurance programs. Data from the 7 states fell into 3 periods: recent (2012), mid-period (2002–2008), and early years (1999). Working backward from the most recent data, several key points follow. First, among behavioral diagnosed young people < age 18, continuously enrolled for 90 or more days, 39.5% of psychotropic medicated youth (*N* = 29,909/75,639) had 2 or more classes overlapping for 90 or more days, and the percent rose to 62.6% for foster care enrollees (18). Examples of 3 drug classes were given but summary data on 3, 4, and 5 or more drug combinations would have identified the size of populations on complex regimens which lack robust evidence that benefits outweigh risks. Such data would compel action for research on widely used off-label combinations of marketed medications, e.g., in large simple trials in community treated populations. Comparison with the 1999 pioneering data of Martin et al. (32) is limited by design and overlap rule differences but it seems clear across 20+ years that polypharmacy in Medicaid populations grew significantly among large proportions of psychotropic treated youth. In addition, documenting long exposures to medication in youngsters highlights the issue of unknown risks to developing youth. Second, 2002–2008 trends in continuously enrolled <18-year olds with any psychotropic dispensing showed substantial growth (19.8–27.3%) by 2008 in 3 or more within or inter-class regimens—primarily (>80%) in interclass rather than within class for 22.3% of foster care medicated youth (33). These data yield a clear pattern of growth of complex regimens in the 2000s compared with earlier years. Third, quality assurance efforts can be useful. Essock et al. analyzed a cohort of psychotropic medicated youth on 4/1/2008, 12.7% had 3 or more psychotropic classes for 90 or more days which was triggered by a flag for a “questionable” clinical prescribing practice based on expert advisory committee consensus (34). For full impact, a follow up comparison study would establish the value of monitoring questionable practices at the state level. In a somewhat similar fashion, Medhekar et al. (35) assessed the impact of physician specialty (psychiatry or primary care) on polypharmacy in a southern state managed care population (*N* = 24,147). The findings on polypharmacy (2 or more classes for 60 or more days) were 5.3 and 3.6 times more likely for single or multiple providers that included psychiatrists.

**TABLE 2b:** Single/two state medicaid pediatric psychotropic polypharmacy studies using state-based data.

**Data source, Study period, References**	**Age, yrs**.	**Other**	**Psychotropic concomitants**	**Overlappingdays**	**Outcome**
Kentucky Medicaid, 2012–2015, Lohr et al. (18)	<18	*N* = 273,393, continuously enrolled w/ behavioral diagnosis across 4 years	≥2 inter-class	≥90	39.5% of the cohort had ≥2 inter-class concomitants for 90 or more days. 57.2% had 2 classes; 10.2–13.4% had 3 classes for ≥90 days.
Connecticut, 1999, Medicaid, Martin et al. (32)	<19	*N* = 9,447 with any psychotropic dispensing	≥2 inter-class	≥7	Among those with a psychotropic dispensing, 13.6% had 2 or more classes concomitantly.
Ohio Medicaid, 2002–2008, 7 year trends, Fontanella et al. (33)	<18	*N* = 26,252–50,311, continuously enrolled w/ any psychotropic dispensing	Any ≥3 classes, within or inter-class by eligibility group	Codispensed: 1) any meds 2) inter-class	1) ^*^FC: 19.8–27.3%; ^**^SSI 18.0–24.9%. 2) ^*^FC: 17.0–22.3%; ^**^SSI 14.3–19.5%, illustrates that interclass is more prevalent than within class polypharmacy.
New York, point prevalence, Essock et al. (34)	<18	*N* = 46,828 Prescribed psychotropic classes on 4/1/2008, w/ >90 days duration	≥3 inter-classes defined as clinically questionable	≥90	12.7% of 25,727 had long use (>90 days) of ≥3 psychotropics that triggered a flag for questionable practice by expert advisory board.
Texas Managed Care, 2013–2015, to assess single/multiple providers associated w/ pediatric psychotropic polypharmacy, Medhekar et al. (35)	<19	*N* = 24,147 w/ single or multiple prescribers and a mental health diagnosis	≥2 inter-class	≥60	20.1% of youth had 2 or more psychotropic classes. Patients with a psychiatrist involved in the treatment had 5.3 and 3.6 times higher odds of receiving polypharmacy as single or multiple prescribers, respectively.
2 abutting mid-Atlantic states, 1999, Medicaid & SCHIP, dosReis et al. (36)	<20	*N* = 8,953 (State A); 48,080 (State B), any continuously enrolled	≥2 inter-class within same month	Duration of overlapping months	Any months of 2 or more classes for State A (27.9%) and State B (29.7%); 5–12 months of concomitant use for A (43.2%) & B (37.5%).
Mid-Atlantic Medicaid,2014, Zito et al. 2020 (20)	<20	*N* = 237,393, continuously enrolled w/ any antidepressant dispensing	ATD + 1 class; ATD + 2 classes; ATD+ ≥3 classes	≥60	^***^ATD + 1 class=22.1%; ATD + 2 classes=14.2%; ATD+ ≥3 classes=5.65%. 25% of ATD-medicated youth had a behavioral diagnosis. Examples: ATD + ATP, ATD+stimulant, and ATD+α-agonist.

* *FC, Foster Care;*

** *SSI, disability insured;*

*** *ATD, Antidepressant*.

### Polypharmacy in Privately Insured Populations

Do public and private polypharmacy patterns differ? This compelling question arises from earlier analyses of antipsychotic use comparing prevalence from Medicaid and privately insured youth (54). Crystal et al. compared findings from separate studies of public and private insurance data and reported a roughly 5-fold greater proportion of youth with antipsychotic use in poor and vulnerable youth than in privately insured youth (54). In the present study, no direct comparative analysis of polypharmacy between public and privately insured youth was identified. Opportunities from federal survey data are limited to point prevalence data (16). For polypharmacy, comparisons are difficult partly because of limited access except broadly from separate studies of data sources (7, 54). In general, greater polypharmacy patterns are expected in publicly insured than privately insured youth. Federal oversight policies (11, 12) support the inference. Fuller discussion of the discrepant patterns are beyond the limits of this paper.

Three striking factors from Table 3 studies include the following. First, the two most recent studies by the same team used Market Scan data, featured off-label concomitant use for ADHD and were industry funded (37, 38). In the earlier study, the authors analyzed data separately for children and adolescents with a diagnosis of ADHD alone or with comorbidities and with a stimulant dispensing. The outcome for 6–12-year olds showed stimulant plus 2 or more medications affected 35.3% of those with ADHD with comorbidities and 13.3% of non-comorbid ADHD diagnosed children. The later study (37) followed similar criteria and found slight increases in concomitant use, emphasizing the use of common off-label combinations of stimulants and selective serotonin reuptake inhibitors (SSRIs) or second-generation antipsychotics. While a number of studies have profiled ADHD diagnosed polypharmacy [(26), Table 1; (17), Table 4], the comparisons are limited by varying study populations, age groups, design, overlap rules and the precision of the outcome itself. Second, Bali et al. analyzed IMS LifeLink data to address a very specific question on the combination of a long-acting stimulant with a subsequent antipsychotic in the follow-up year (39). Only 3.9% of 37,981 had an antipsychotic added in the follow-up year. Attributing the 71-day longer persistence of the concomitant users as a benefit to adherence is questionable. Third, the earliest privately insured polypharmacy study (40) was unique in presenting survey data from volunteer psychiatrist members of the American Psychiatric Association. Because the data on 332 youth managed by 189 treating psychiatrists originated at physician offices, a precise profile of psychotropic medication treatment was possible: monotherapy (40%); 2 concomitant medications (30.5%); 3 concomitant medications (10.2%); 4 or more medications (2.9%), and no medication (16.2%). The data were collected in 1997 and 1999 and findings from a later Medicaid source support patterns of polypharmacy in psychiatric specialty care exceeding that of primary care (35).

**TABLE 3:** Pediatric psychotropic polypharmacy in privately insured populations.

**Data source, Study period, References**	**Age, yrs**.	**Other**	**Psychotropic concomitants**	**Overlappingdays**	**Outcome**
2011–2014 Truven Market Scan, Zhou et al. (37)	6–17	133,354–157,303 children;95,632–111,280 adolescents.ADHD alone or w/comorbidity, Continuously enrolled w/ ≥1 stimulants	Any 2,3,4, ≥5 concomitant medications	≥30	Stimulant + 1 or more medications increased for: Children: 22.9–25.0%; Adolescents: 25.2–28.2%. Off label: stimulant + ^*^SSRI; stimulant + ^**^AAP were common
2009, Truven Market Scan, Betts et al. (38)	6–17	*N* = 71,201 children 6–12;*N* = 49,959 adolescents 13–17. ADHD alone or w/ comorbidity and stimulant use	Stimulant + 14 other class pairs within & interclass	≥30	12.6% of non-comorbid ADHD had ≥2 classes while 41.7% of ADHD with comorbidities experienced combinations. ^*^SSRIs and ^**^AAPs were common.
2004–2006, IMS LifeLink, Bali et al. (39)	6–16	*N* = 37,981 long-acting stimulant users w/ 1 year followup for antipsychotic users	^***^LAS w/ or without concomitant antipsychotic	≥14	Only 3.9% of LAS users had a concomitant antipsychotic added. 71 day greater persistence in the off-label combination was deemed improved adherence compared with LAS alone.
1997–1999 surveys of ^****^APA member volunteers, Duffy et al. (40)	2–17	189 prescribing psychiatrists for 332 youth	2; 3; ≥4 within or interclass	Point prevalence	40% monotherapy; 30.5% 2 medications; 10.2% 3 medications; 2.9% ≥4 medications, 16.2% no medication prescribed.

* *SSRI, Selective Serotonin Reuptake Inhibitor;*

** *AAP, Atypical Antipsychotic;*

*** *LAS, Long-acting stimulant;*

**** *APA, American Psychiatric Association*.

**TABLE 4:** Pediatric psychotropic polypharmacy in diagnosed populations.

**Data source, Study period, References**	**Other**	**Age, years**	**Diagnosed population**	**Psychotropic concomitants**	**Outcome**
Southern state, 1996–2005, McIntyre and Jerrell (41)	Cross-sectional	0–17	*N* = 1,544 w/ Depression diagnosis, continuously enrolled 9/12 months	≥2 psychotropic medications	Polypharmacy increased from 6.7% (1996) to 41.6% (2005)—a 6-fold increase & is largely off-label. Polpharmacy increased with increased comorbidity.
US privately insured, 2001–2009, Spencer et al. (21)	Polypharmacy prevalence	<20	Autism Spectrum Disorder (ASD), w/ ≥6 months continuous enrollment, *N* = 33,565	≥2 or ≥3 classes with ≥30 days overlap	Among the diagnosed cohort, 35% had ≥2 classes, 15% had ≥3 concomitant classes. The median duration of polypharmacy was 346 days.
MAX, 2001, 50 states + D.C., Mandell et al. (22)	Polypharmacy prevalence	<21	Autism Spectrum Disorder, *N* = 60,641 with ASD diagnosis & psychotropic rx	≥3 medications with ≥30 days overlap	20% of foster care psychotropic medicated youth had ≥3 concomitants among 6 psychotropic classes compared with 7% for poverty subgroup and 11% with disability status.
MAX, 41 states,2000–2003, Schubart et al. (42)	4 year trend analysis, x-sectional	3–17	*N* = 12,843–18,562 with Autism Spectrum Disorder diagnosis	≥60 day overlap for pairs of psychotropics	26–30% had pairs in 6 groupings.
Southern State ASD treatment program,2000–2008, Logan et al. (43)	Polypharmacy prevalence	8	The state is part of a CDC Autism Spectrum Disorder surveillance program. *N* = 629	Six 2-class combinations with ≥30 days overlap	Among the 60% (~377) with a dispensed psychotropic, 41% (~150) had 2-class combinations. Unfortunately, the extent of 3 or 4 or more class concomitant use is not known.
MAX, 28 states, 1999–2006, Winterstein et al. (17)	1–5 year follow up (f/u) study	0–17	Attention Deficit Hyperactivity Disorder (ADHD), 3–18 years old, *N* = 16,626 w/ f/u for new users of stimulantc.	≥3 classes, any days overlap	Psychiatric polypharmacy of ≥3 classes increased from 8.5% (year 1) to 13.4% (year 5) for children 3–9 years old at initiation of stimulant. Any ≥3 classes in a subsequent year affected 25.35%.

### Polypharmacy in Diagnosed Populations

The goal of polypharmacy research is enhanced when clinically meaningful designs are chosen. Among the six studies assessing a clinically diagnosed population, several findings stand out. First, depression comorbidities increased exposure to polypharmacy (41). The growth of comorbidities is, in itself, on the rise (47, 55) and are beyond the present review. McIntyre and Jerrell examined 1996–2005 trends, which occurred during the decade that covered the dramatic time when a meta-analysis of antidepressant (ATD) pediatric clinical trials showed a significant association with suicidal thoughts (56). That provocative study led to the FDA boxed warning on the official antidepressant label and subsequently reduced ATD prevalence in practice. The reduction was most prominent for younger aged children and least for those diagnosed with major depressive disorder (57). Analyzing data from 1,544 younger than 18-year olds in a southern state, McIntyre and Jerrell examined antidepressant polypharmacy in a 24 month follow up of new antidepressant users. By removing switching of antidepressants, the authors identified polypharmacy of 2 or more psychotropic medications which rose dramatically from 6.7% (1996) to 41.6% (2005). The authors identified this decade as “epochal” in the growth of inter-class polypharmacy as common practice. Second, four studies investigated polypharmacy among youth diagnosed with autism spectrum disorders (ASD) (21, 22, 42, 43). These studies cover a considerable time period (2001–2009) yet provide little consistency because of differences in the age of youth selected, number of overlapping days selected, and the imprecise polypharmacy outcome. Also, the value of restricting outcomes to pairs of classes is unclear as the extent that pairs are part of 3 and 4 or more class concomitants is unknown but hides the increased risk of drug interactions and the wider range of adverse drug events for more complex regimens (42). Third, a rough comparison made between public and privately insured populations suggests that the use of 3 inter-class concomitant regimens are similar in some studies, 15% privately insured and 20% publicly insured (21, 22). Lastly, Winterstein et al. provide a clinically rich study designed to assess 3 or more class polypharmacy in the 5 years following an initial stimulant dispensing with 25.3% receiving a 3-class regimen at least once in a subsequent year (17).

### Polypharmacy in the Foster Care Population

Table 5 confirms a well-established fact, namely that foster care youth are likely to be exposed to polypharmacy in many times greater proportions than their non-foster peers as documented by Government Accounting Office studies (11, 12). Several points can be made from studies shown on Table 5. First, two single state Medicaid studies found there was a 5-fold greater proportion of foster care users of inter-class concomitant regimens than their non-foster care Medicaid peers (44, 45). In the study with the latest data (2016), Keast et al. reported outcomes less precisely, i.e., 2–3 or more and 4–5 or more (44) which limits opportunities for comparisons. Second, Raghavan et al. (46) present useful clinical information on a cohort of 403 17-year olds aging out of foster care in a Midwest state. One-third of patients in the cohort who would be aging out of foster care were receiving 3, 4, or 5 concomitant psychotropics. The likelihood that they would make a smooth transition to other health coverage is not known, but the risk associated with abrupt discontinuation of potent combinations is known (58). Third, in terms of precise outcomes, several studies provide exact percentages on inter-class concomitant use.

**TABLE 5:** Pediatric psychotropic polypharmacy in the foster care population.

**Data source, Study period, References**	**Other**	**Age, years**	**Population**	**Psychotropic concomitants**	**Outcome**
2016, soutwestern state Medicaid, Keast et al. (44)	Foster care vs. non-foster care, x-sectional	<21	*N* = 9,325 foster care; *N* = 639,868 non-foster care	≥2 interclass for ≥90 overlapping days	9.2% concomitant use in foster care vs. 1.9% in non-foster care youth. As a percent of foster care psychotropic medicated youth, 41.3% had ≥2 classes: 35% w/ 2–3 classes and 6.3% w/ 4–5 classes.
Medicaid drug utilization oversight program, Colorado DUR (45)	Compare 2012 and 2015 foster care polypharmacy	<18	*N* = 16,789 foster care; 406,124 non-foster care.	≥2, ≥3, ≥4 interclass for ≥60 overlapping days	In 2015, 26% of foster care youth received one or more psychotropic classes, roughly 5 times greater than non-foster care; 7% received ≥2; 2% received ≥3; <1% received 4 or more. Similar pattern 12 &15.
Midwest state Medicaid, Dec 2001–May 2003, face to face surveys, Raghavan et al. (46)	To assess medication patterns in a cohort aging out of foster care	17	*N* = 403, Participants self-reported medication use in past month	2,3,4,5,7 concomitant classes	*N* = 146 with any psychotropic medication; 2 concomitant (47); 3 concomitant (22), 4 concomitant (18); 5 concomitant (7). ~One-third of medicated youth had 3, 4, or 5 or more concomitant psychotropics.
2002–2007, 47 states + D.C., 6 year trend, Rubin et al. (24)	State-specific polypharmacy prevalence	3–18	Foster care, continuously enrolled & with antipsychotic dispensing, *N* = 686,080	≥3 class for≥30 overlapping days	Wide variation in 3 class polypharmacy across states: 0.5%−13.6% had ≥3 classes, one of which was antipsychotic.
Southeastern state2003–2008, Brenner et al. (23)	Community intervention trial of “treatment foster care”	2–21	*N* = 240, parent-reports at baseline of intervention program	2, 3, or ≥4 psychotropics; point prevalence	Of the psychotropic medicated youth, 35% had 2 medications; 15.% had 3 medications and 9.2% had ≥4 concomitant medications.
Southeastern state, 2004, foster care population, Zito et al. (15)	Polypharmacy Prevalence	<20	*N* = 472 medicated youth in a random month	Manual review of overlapping dispensings of2, 3, ≥4 classes	Of foster care youth w/ any psychotropic dispensing, 31.1% had 2 concomitant classes; 25.4% had 3 concomitant classes; and 15.9% had ≥4 concomitant classes.

**TABLE 6:** Pediatric psychotropic polypharmacy in special settings.

**Data source, Study period, References**	**Other**	**Age, years**	**Population**	**Psychotropic concomitants**	**Outcome**
Mid-Atlantic state continuity of care outpatient program Wu et al. (48)	Quasi-experimental program evaluation	3–21	*N* = 496, continuously enrolled for 1 year pre, during and post-intervention	≥3 classes w/ ≥15 day overlap	Compared psychotropic polypharmacy of youth enrolled in continuity of care program (≥90 days) with propensity score matched youth in usual care. Polypharmacy did not significantly differ between groups, affecting 29 to 31 to 21% across 3 years.
A state residential treatment center vanWattum et al. (49)	prevalence of polypharmacy change, admission to discharge	11–18	*N* = 131, Admission to discharge change in polypharmacy	≥2 psychotropic medications	Discharged youth had fewer polypharmacy treated youth and 60% increase in the non-medicated subgroup.
Juvenile secure facility, 1 year, 2007–2008 Lyons et al. (50)	Change in polypharmacy, admission to discharge	12–22	*N* = 668; 68 with psychotropic medication	≥2 psychotropic medications in the same month	There were 10.2% medicated within 1st month of admission; 48.5% received ≥2, with atypical antipsychotics and antidepressants most common.

Rubin et al. (24) analyzed state-specific concomitant regimens of 3 or more classes making clear the wide range of findings across 44 states from 0.5 to 13.6%, many including an antipsychotic medication. Several assessments had precise outcomes but did not eliminate switching by using a point prevalence overlap (23) or up to 30 days overlap (15).

### Polypharmacy in Special Settings

The last group of papers pertains to program evaluation (48) to reduce polypharmacy in Medicaid outpatients and 2 studies in restricted settings (49, 50). Three findings these studies emphasize are: First, publication of peer reviewed assessments of public programs is critical for accountability on treatment of vulnerable or restricted populations and lends strength to quality improvement efforts. This is particularly true when youth status is involuntary and there is a potential for punitive action. Second, the extensive use of antipsychotics in this and other studies of complex regimens highlights the need to evaluate the role of psychotropic drugs for disruptive and aggressive behaviors. The limited interest by federal agencies in assessing medication treatment of childhood aggression essentially amounts to turning a blind eye for more than 20 years, which indirectly contributes to the growth of second-generation antipsychotics for behavior disorders. The TOSCA study is an exception (59) but the findings indicated that although adding risperidone to a long-acting stimulant produced some initial improvement at 9 weeks, the combination was deemed only moderately more effective than placebo. At 1 year, active drug and placebo group treatment differences were not apparent. The authors called for more research on this question and why the combination is widely prescribed. Third, restricted populations may age out of their insurance coverage and, upon discharge, experience abrupt discontinuation with potentially severe withdrawal syndrome. As the Raghavan et al. (46) cohort of youth aging out of foster care illustrated (Table 5), 37% of foster youth will leave publicly funded care with 2–5 concomitant psychotropic medications and uncertainty about follow up health insurance coverage. It is not known if comprehensive treatment planning will assure transition to new coverage in a timely way to avoid drug withdrawal.

## Discussion

Despite the wide range of criteria in the design of the studies reported above, several points are clear. First, pediatric psychotropic polypharmacy affects substantially more children and adolescents today than was the case 20+ years ago. As many as 300,000 youth received 3 or more classes concomitantly in 2011–2016 (19). Second, the duration of concomitant use is relatively long, e.g., 69–89% of annual medicated days (31). Third, adverse event reports were associated with more complex regimens (3-class compared with 2-class concomitant regimens (27). In another study, increased depression comorbidities were associated with more complex polypharmacy (41). These findings raise questions about the long-term effectiveness and safety of off-label combinations as well as the relationship of multiple comorbidities to overprescribing. At the core of pediatric psychotropic prescribing lies a deeper question about the U.S. standard of medical care for the off-label treatment of behavioral problems of children and adolescents, a topic beyond the scope of this review.

We acknowledge the limitations of this review. First, some studies may have been missed as titles and abstracts do not always provide critical data on inter-class polypharmacy. Second, some studies combined same class and inter-class polypharmacy and we chose to include them to illustrate that inter-class regimens are the greater proportion of affected youth. Overall, the trends are clear, although study designs are varied and metrics are imprecise so that their implications can be missed. Nonetheless, we appreciate that some studies demonstrate clear, complete and precise profiles of prescribing patterns (19, 24, 40, 46).

The decision to limit analysis to U.S. studies was based on the authors' knowledge of the literature broadly in the past 30 years. U.S. medication prescribing and usage is generally regarded as more intensive than in other western countries. A 2015 review of international pediatric pharmacotherapy by a leading European scholar makes the point that pediatric psychotropic use is “many times more in U.S. than in all other countries” (60). In one example of a polypharmacy review from Europe, there were few European papers with a claims analysis (61).

In the following sections, we attempt to broaden the discussion to several implications of the growth of pediatric psychotropic polypharmacy.

### Why Are 3 or More Inter-class Pediatric Psychotropic Regimens Increasing?

#### Biopsychosocial Model Is Ignored

In the 43 years since psychiatrist George Engel called for a new medical model in a biopsychosocial framework (62), his model has been overtaken by the biological psychiatry model (63). Many reasons have been identified for failing to fully integrate non-pharmacologic therapies (workforce, insurers, insufficient family time) or to not fund community-based alternatives. No doubt, these are formidable challenges and will take a massive commitment from multiple stakeholders (academic research, government authorities and funders, and prescribing physician societies) to reform the system. Stakeholder silence has led to further reliance on pills—even for social determinants of poor child behavior such as poor family stability, unsafe schools, and shelter living. Like the cobbler who responds to every problem as a shoe problem, when society asks medicine to relieve social ills, we get prescriptions. After analyzing more than 20 years of data and at least 35 studies on psychotropic polypharmacy, the prescriber's response that “This is all I have” seems woefully inadequate.

#### Pharmacologic Assumptions Are Not Valid

Accepting the appropriateness of complex, off-label regimens in the pediatric population may reflect various beliefs. First, the efficacy from individual drug trials may be assumed to be cumulative across classes of concomitants and will not be exceeded by the collective adverse events. Hilt et al. (27) illustrated the fallacy of this assumption, as did Turner et al. (52). While this assumption is sometimes justified for serious emotional and mental disorders, e.g., schizophrenia, it is difficult to justify for behavioral conditions, e.g., ADHD without comorbidities (17, 25, 26, 37).

In addition, complex combinations increase the risk of drug-drug interactions. Drug-drug interactions among 3-, 4-, or 5 or more classes is mathematically far more complicated and there is relatively little work in this area for pediatric psychotropic combinations (64). For a common example likely to be found in some youth, the combination of an SSRI and a second-generation antipsychotic in long-term concomitant regimens has been shown to produce blockade of P-450 enzymes caused by competitive inhibition of the enzymes (64) and could lead to a serotonin syndrome or to toxic levels of an antipsychotic. An adult study analyzed pharmacoepidemiologic data from Scottish adults across all medications for medical and mental conditions (65). Comparing 1995 with 2010, the authors found a nearly 3-fold increase in risk of a potentially serious drug-drug interaction among adults receiving a CNS drug (1.2–3.4%) (65).

Adverse events from polypharmacy combinations may be difficult to distinguish from new behavioral symptoms and lead to more medications (66). Furthermore, the evidence of the effectiveness and safety of concomitant regimens is often assumed to be adequate. However, the published literature does not support that assumption. Pediatric clinical trials of concomitant use are criticized for weak designs (67) and haven't improved much.

#### Post-marketing Evidence Is Ignored

Effectiveness studies of second-generation antipsychotics (SGA) have failed to show superiority over first generation products as demonstrated for children diagnosed with early-onset schizophrenia and schizoaffective disorder in the TEOSS study (68). In addition, SGAs can lead to new, serious adverse drug events e.g., treatment emergent diabetes (9, 69). A sobering post-marketing picture has emerged in the 25 years since SGAs were introduced (70). The ethical decisions that support SGA use for severe emotional and mental disorders, e.g., schizophrenia are largely based on severity and relief of suffering but are in stark contrast to the less justifiable use of atypical antipsychotics in combination with a stimulant and antidepressant in ADHD diagnosed youth. These off-label combinations lack robust evidence that the benefits outweigh the risks. Similarly, there is strong concern voiced about the use of SSRIs for the treatment of children (71) both in terms of weak efficacy, biased maintenance research studies, and on the alarming uncertainty that benefits exceed risks (72, 73).

The FDA is a stakeholder of great importance in creating new knowledge on approved medications. Phase 4 of the FDA drug development model constitutes the post-marketing phase when new information about a drug's effectiveness and safety in large populations of community treated persons could be analyzed. Wider usage potentially will reveal new knowledge that the proprietary trials conducted for FDA approval were not powered to reveal. Post-marketing effective studies can provide support for off-label pediatric drug use (74). It is not clear why the drug development graphic on the FDA website has changed over the years to one that only emphasizes safety for (phase 4) post-marketing research rather than for both effectiveness and safety.

At the broadest level, the low value of healthcare procedures with unknown effectiveness but with known risk of harm deserves attention (75). In this thoughtful commentary, Brownlee and Korenstein provide an analysis applicable to the unnecessary use of off-label medications for the mental and behavioral treatment of youth. They suggest “…the failure to focus greater attention on the physical and psychological harms of overuse has hampered efforts to reduce it,” resulting in resistance to calls to rein in overprescribing.

### New Developments in the Prescribing Practice Literature Could Reduce Unnecessary Polypharmacy

In the past decade, pediatric clinical researchers have begun to create protocols to support the needs of clinicians who “inherit” new patients with complex regimens that the clinician may view as excessive or pose challenges to careful management (76). Adapting the methods of geriatric pharmacology, “deprescribing” is slowly growing in importance to address mental health prescriber needs (77), probably an indirect consequence of the ever-growing use of complex concomitant regimens. A recent survey of primary care and psychiatry clinicians in community public health centers focused on overprescribing and respondents acknowledged concerns about complex drug regimens in children but suggested resources are needed to support deprescribing (78). An additional concern relates to the patient experience of problems to successfully discontinue psychotropics. The problems of adults with difficulties discontinuing benzodiazepines are joined by more recent concerns on the withdrawal syndrome associated with SSRIs (79). When youth who are seen by multiple clinicians and not known well by any clinician, it is easy to understand the skepticism of some clinicians that SSRIs are hard to discontinue. Indeed, a separate literature on patient-focused medication problems has emerged (80).

Concerns about overdiagnosis and overtreatment have been articulated by non-US academic psychiatrists (81) and by dissenting U.S. leaders (82). Within the U.S. psychiatric community, Steingard's recent book, *Critical Psychiatry*, elucidates controversies related to the Diagnostic and Statistical Manual (DSM-5); deprescribing; and the role of the pharmaceutical industry in creating biased analyses for their heavily promoted, initially costly new products (83). Such critical discourse parallels the growing disappointment with clinical experience over decades, for example, described by Rosenheck as “irrational exuberance” for antipsychotic use (70). The problem is particularly acute with respect to children where widespread adoption of second-generation antipsychotics for non-psychotic youth in complex regimens is evidenced in the tables above. While adoption of SGA antipsychotics has been trending downward) in publicly insured youth (7, 13, 14), oversight of inter-class polypharmacy and research on it is far less prominent.

### Research Funding

The clamor for effectiveness research in the studies reviewed above is remarkable; many authors ended their discussions with firm calls for research to establish the effectiveness, safety and tolerability of complex concomitant regimens in community-treated populations. In light of the weak or absent evidence for widely used combinations of second-generation antipsychotics and antidepressants in youth, large randomized simple trials or other post-marketing effectiveness research in community populations should be prioritized for public funding (53). Several regional academic sites with electronic health records could follow randomized trial protocols with consenting patients to evaluate response to less complex regimens against usual treatment.

We join the call seeking federal and foundation funding for deprescribing research (78, 84). Also, we urge robust responses to the request for proposals from the Patient Centered Outcomes Research Institute (PCORI) for large simple trials. Large simple trials with a patient-centered focus especially fit the need to establish the benefits and risks of complex concomitant regimens that will be acceptable and tolerably consumed by youngsters in community treated populations.

## Conclusion

A review of 20 years of pediatric psychotropic polypharmacy supports standardizing criteria in the design of population-based studies so as to maximize information on the number of youth receiving regimens of 3-, 4-, and 5 or more concomitant classes and the duration of such use. Calling together leadership in mental health services, child psychiatry and pediatrics would kickstart this effort in the hope of generating a clinical call for post-marketing research to address the effectiveness, safety and tolerability of complex drug regimens in youngsters.

## Author Contributions

JZ: supervised the literature search and analyzed 35 studies for inclusion, and wrote and revised the draft. YZ: conducted the computerized search and read revised drafts. DS: reviewed drafts, edited text, and collaborated on content of discussion. All authors contributed to the article and approved the submitted version.

## Conflict of Interest

The authors declare that the research was conducted in the absence of any commercial or financial relationships that could be construed as a potential conflict of interest.
